# Perspective: The Mechanobiology of Hepatocellular Carcinoma

**DOI:** 10.3390/cancers13174275

**Published:** 2021-08-25

**Authors:** Abigail E. Loneker, Rebecca G. Wells

**Affiliations:** 1 Department of Bioengineering, University of Pennsylvania, Philadelphia, PA 19104, USA; loneker@seas.upenn.edu; 2 Physical Sciences in Oncology Center PSOC@Penn, University of Pennsylvania, Philadelphia, PA 19104, USA; 3 Center for Engineering MechanoBiology, University of Pennsylvania, Philadelphia, PA 19104, USA; 4 Department of Medicine, University of Pennsylvania, Philadelphia, PA 19104, USA

**Keywords:** liver, stiffness, nuclear deformation, lipid droplet

## Abstract

**Simple Summary:**

Hepatocellular carcinoma (HCC) is a deadly primary liver cancer that most often develops in a cirrhotic (highly scarred) liver. Cirrhosis is associated with large-scale mechanical changes in the liver, with increases in stiffness to levels that alter liver cell behavior. Importantly, recent research has yielded two key observations: that mechanical changes in the microenvironment can promote the development and progression of a variety of cancers, and that nuclear deformation can increase genetic instability and the accumulation of DNA damage in some contexts. HCC is a unique cancer given that it occurs in an environment that is already very stiff and that liver cells in cirrhosis have highly deformed nuclei. This suggests that mechanical changes in the liver could be a source of genetic instability that leads to cancer development.

**Abstract:**

Hepatocellular carcinoma (HCC) is the second most deadly primary cancer in the world and is thus a major global health challenge. HCC primarily develops in patients with an underlying chronic liver disease, the vast majority with advanced cirrhosis, characterized by increased matrix deposition and liver stiffness. Liver stiffness is highly associated with cancer development and poor patient outcome and is measured clinically to assess cancer risk; cirrhotic livers greatly exceed the threshold stiffness shown to alter hepatocyte cell behavior and to increase the malignancy of cancer cells. Recent studies have shown that cirrhotic liver cells have highly irregular nuclear morphologies and that nuclear deformation mediates mechanosensitive signaling. Separate research has shown that nuclear deformation can increase genetic instability and the accumulation of DNA damage in migrating cancer cells. We hypothesize that the mechanical changes associated with chronic liver disease are drivers of oncogenesis, activating mechanosensitive signaling pathways, increasing rates of DNA damage, and ultimately inducing malignant transformation.

## 1. Introduction

Hepatocellular carcinoma (HCC) is the second most common cause of cancer death in the world, resulting in up to 800,000 deaths per year [1], and is one of the few cancers that is increasing in incidence [1]. Cirrhosis and increased liver stiffness are significant risk factors for the development of HCC, with 80–90% of cases occurring in stiff, cirrhotic livers [2]. Furthermore, the magnitude of liver stiffening is highly correlated with patient outcomes, such that patients with the stiffest livers have the highest risk of developing HCC, the highest mortality rates, and the shortest survival times [3,4,5]. The association between increased stiffness and HCC has been documented in patients with a variety of underlying etiologies, and stiffness is used for clinical assessment [6]. Stiffness compromises important hepatocyte functions [7,8,9] and multiple mechanosensitive pathways have been implicated in HCC progression. The published research thus shows a strong association between abnormal tissue mechanics and the development of HCC.

There is now a growing body of mechanobiology literature (as detailed in the rest of this volume) suggesting that tissue stiffness contributes to malignancy and modulates the behavior of multiple cell types, including hepatocytes [9,10]. It remains unknown, however, whether liver stiffness plays a causative role in HCC oncogenesis, rather than just being correlated with the progression of chronic liver disease. In this perspective, we review general lessons from the field of cancer mechanobiology and argue that HCC is a mechanically-driven primary cancer, occurring in livers characterized by large shifts in cell and tissue mechanics that lead to changes in cell behavior and the accumulation of DNA damage. We emphasize that better understanding the impact of mechanical stress on liver cells has the potential to lead to new interventions to prevent HCC development.

## 2. The Liver Progressively Stiffens in Fibrosis and Cirrhosis

The development of HCC is closely associated with the progression of fibrosis and cirrhosis. Cirrhosis, which is the accumulation of extracellular matrix (ECM) proteins and the disruption of normal liver architecture, arises in the setting of chronic liver disease, including viral hepatitis, alcoholic and nonalcoholic fatty liver disease (NAFLD) and cholestatic diseases. The relationship between liver stiffness and fibrosis is complex. In early stages, stiffness increases out of proportion to fibrosis, largely due to increases in lysyl oxidase (LOX)-mediated collagen cross-linking [11,12]; the increased stiffness enables the first round of myofibroblast differentiation and abnormal matrix deposition. A similar sequence of events has been reported for the development of lung fibrosis [13]. Even at later stages, liver stiffness and collagen content are not linearly related, although the progression of fibrosis is highly correlated with increasing liver stiffness, both in rodents [11,14] and human patients [15,16]. Stiffness measurements have proven to be highly predictive of fibrosis progression in patients with liver diseases of multiple etiologies and, due to the accuracy and noninvasive nature of screening, transient elastography and magnetic resonance elastography (MRE) are now frequently used clinically for disease surveillance and for assessing prognosis [6,17,18].

Inflammation is also a major component of most chronic and acute liver diseases and may be both a cause and effect of increased liver stiffness. The activation of hepatic stellate cells is a crucial event in hepatic inflammation and early fibrosis, and liver stiffness has been shown to trigger this activation, even before increased matrix deposition [16]. Interestingly, even in the case of acute liver failure, liver stiffness, cell damage and hepatic stellate cell activation are positively correlated, suggesting that inflammation may lead to increases in tissue stiffness [19,20]. A similar relationship has been shown for patients with hepatitis C, where increased inflammation (as measured by serum alanine aminotransferase (ALT)) correlates with liver stiffness among patients with early fibrosis [21].

In addition to increasing in bulk stiffness, the liver demonstrates more heterogeneity in stiffness as fibrosis progresses [9,22]. Normal mouse livers exhibit a modest level of mechanical heterogeneity, with portal tracts being slightly stiffer than pericentral regions, both in the range of ~100–300 Pa [9]. The presence of fibrotic septa in these mouse livers dramatically increases local stiffness by more than a factor of 10, but this effect does not propagate to areas distant from collagen deposition. Nonetheless, these regional mechanical changes are likely to have an impact on the phenotype of nearby cells, as described below. The presence of a stiffness gradient may also impact cell behavior, as in vitro experiments have shown that cells will migrate towards higher stiffness areas [23].

**Figure 1:**
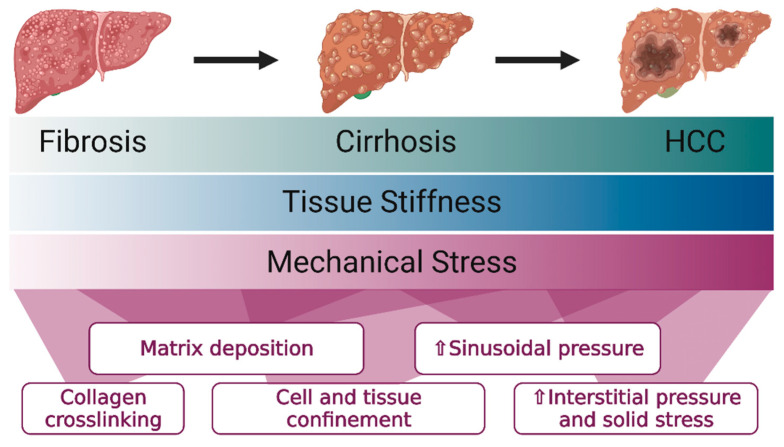
Tissue stiffness and mechanical stress compound over time as fibrosis progresses to cirrhosis, even before developing HCC.

Other features of the fibrotic and cirrhotic liver also have an impact on liver mechanics, although these have not been as well defined as changes in bulk stiffness. For example, the presence of portal hypertension increases pressure in the sinusoids, potentially compressing and mechanically stressing liver cells. This is particularly relevant in the context of the thickening and stiffening of the capsule that occurs in advanced liver disease [24], which further constrains tissue expansion and impacts the mechanical environment that liver cells experience. Fibrotic tracts can also fully encase liver cells, generating stiff boundaries and increasing pressure on individual lobules. Both solid stress and interstitial fluid pressure (referring to the compressive and tensile forces exerted by the solid and fluid components of the liver [25,26,27,28]) are likely to change significantly in cirrhosis due to the accumulation of various matrix components. This has already been shown for pancreatic adenocarcinoma, where collagen and hyaluronic acid deposition correlate with large increases in interstitial fluid pressure and solid stress [27,29,30]. This phenomenon has primarily been examined in the context of drug delivery, as vessels within the tumor are constricted by increased pressure, preventing the penetration of chemotherapy drugs [31,32,33], however, more recently it has been shown that different stromal morphology in pancreatic cancer correlates to different functional outcomes, suggesting that matrix mechanical changes could alter oncogenesis and cancer progression [34]. Importantly, these mechanical changes do not act on cells in isolation (Figure 1); increased interstitial pressure will compress the matrix, increasing the effective stiffness that cells sense in addition to directly compressing them.

## 3. Liver Cells Are Mechanosensitive

Almost all cells studied thus far are mechanosensitive, and the same holds true for cells of the liver. This has significant implications for the phenotype of cells in mechanically abnormal environments such as fibrosis. For example, hepatocytes remain fully differentiated in vitro only within a narrow window of stiffness [9], dedifferentiating rapidly even with modest increases. High substrate stiffness alters cell morphology, reduces hepatocyte-specific gene expression, and decreases hepatocyte-specific metabolism [9,35]. Elevated shear stresses have been shown to increase the severity of the hepatocyte response to matrix stiffness, leading to dedifferentiation [35], suggesting that multiple mechanical stresses can amplify cell responses. This is especially relevant given that HCC occurs in a cirrhotic environment with numerous mechanical stresses and that cycles of hepatocyte death and regeneration are thought to underly HCC oncogenesis. Furthermore, hepatocytes remain mechanosensitive even in malignancy; elevated liver stiffness increases the proliferation, invasion, and chemotherapeutic resistance of malignant hepatocytes, increasing the severity of HCC [7,8,9].

Portal fibroblasts and hepatic stellate cells are also mechanosensitive, increasing ECM gene expression and matrix deposition in response to mechanical cues in stiff environments [36,37,38]. As noted above, liver stiffness has been shown in rodent models to precede fibrosis, suggesting a mechanism whereby fibrosis is initiated by increases in tissue stiffness through collagen crosslinking [11]. Consistent with this mechanism, blocking early tissue stiffening through the use of LOX inhibitors slows myofibroblast activation and the progression of fibrosis due to CCl_4_-mediated liver injury [11]. Activated fibrogenic cells play an important role in the emergence of HCC. Persistent activation of both hepatic stellate cells and portal fibroblasts promotes their differentiation into highly contractile myofibroblasts, making them likely precursors of cancer-associated fibroblasts (CAFs) in HCC [39,40]. Several CAF-dependent mechanisms promote hepatocarcinogenesis, including alteration of the liver ECM, increased tumor angiogenesis, and the secretion of inflammatory and tumor permissive cytokines [41].

As has been shown for other cancers discussed in this volume, numerous mechanosensitive pathways impact progression of HCC including focal adhesion kinase (FAK), PI3K/AKT [42,43,44], Ras/MEK/ERK [45,46,47], and the transcription factors YAP and TAZ [48,49]. Importantly, these pathways are upregulated in fibrosis [50,51,52,53]. In particular, FAK is more significantly upregulated in cirrhotic tumors than in those with less dense ECM, suggesting that stiffness-dependent FAK activation plays a role in tumor aggressiveness [54]. FAK also plays a key role in activating hepatic stellate cells in early fibrosis, increasing expression of α-smooth muscle actin and collagen [55], both markers of myofibroblasts that are associated with cancer development. Interestingly, FAK depletion in human HCC cells reduces growth by inhibiting histone H3k27me3 [56], consistent with work showing that chromatin condensation can increase DNA damage in epithelial cells under mechanical stress [57]. This suggests that mechanosensitive signaling pathways are activated in early fibrosis, contribute to fibrotic progression, and increase cancer aggressiveness once it develops.

## 4. Mechanically Induced Nuclear Deformation Activates Oncogenic Signaling and Increases DNA Damage

Intriguing recent data have shown that compression and deformation of the nucleus may be significant components of mechanotransduction. Actin stress fibers that form in stiff environments compress the nucleus, permitting the translocation of YAP [58], and increasing instances of nuclear envelope rupture [59]. The same effect is seen if the nucleus is compressed osmotically or in the absence of an intact cytoskeleton, suggesting that nuclear deformation can directly activate mechanosensitive signaling [58,60]. While many of these mechanobiology studies have been completed in mesenchymal cells, recent work analyzing the nuclear morphology of liver cells in cirrhosis suggests that nuclear deformation regulates mechanosensing directly, and that severing cytoskeletal-nuclear links can restore a rounded nuclear morphology and quiescent phenotype [61].

Confined cells (such as those in a cirrhotic nodule) exhibit decreased mitotic fidelity. Mechanical and geometric constraints limit the ability of cells to round up when entering mitosis, generating chromosome segregation defects, multipolar spindles, and asymmetric cell division [62,63,64]. This effect has also been shown in 3D culture conditions, in which cell spheroids embedded in agarose exhibited longer prometaphase periods and spindle misorientation [65], which can contribute to carcinogenesis [66]. The effect of confinement is magnified as the environment stiffens: cells compressed under stiff polyacrylamide gels have more frequent mitotic errors than those under soft polyacrylamide gels [67,68]. Tissue architecture is particularly important for chromosome segregation in the liver. While hepatocytes in a regenerating liver have high mitotic fidelity, those dissociated and expanded on collagen-coated glass coverslips had significantly more lagging chromosomes and a 5-fold increase in aneuploidy [69]. Chromosome missegregation and aneuploidy are common in HCC [70] and may be the result of cell division in a stiff cirrhotic environment.

Importantly, deformation of the nucleus, whether by migration through small pores, direct application of force, or spreading on stiff substrates, has been implicated in the loss of nuclear repair factors and accumulation of DNA damage [71,72,73,74]. Though increased DNA damage from deformation has not yet been documented in primary liver cells, nuclear deformation is increased in chronic liver diseases, raising the possibility that deformation is a mechanism of oncogenesis [61,75]. This may be related to chromatin condensation: in order to prevent mechanically-induced DNA damage, cells may soften their nuclei by decondensing heterochromatin, and preventing decondensation increases double stranded DNA breaks [57]. Cells have been shown to condense chromatin in response to substrate stiffening [76], suggesting that cells in a stiff cirrhotic liver may be particularly sensitive to mechanically induced DNA damage.

Consistent with this idea, recent sequencing of normal and cirrhotic human livers indicates that cirrhosis increases copy number variations and structural variants [77]. While this study did not report mechanical characterization of the tissues that were studied genetically, there is extensive literature (as summarized above) demonstrating that cirrhotic livers are much stiffer than normal or non-cirrhotic fibrotic livers. This is also consistent with work showing that genomic variation (including both somatic mutations and large scale chromosome copy-number variations) scales with tissue stiffness [78]. Previous attempts to explain this phenomenon focused on cell migration as a source of mechanical stress—stiffer tissues have a denser collagen matrix and cells migrating through this matrix experience large scale nuclear deformations that lead to DNA damage accumulation. Given more recent research showing that deformation directly mediates mechanosensing and can induce damage in contexts other than constricted migration [60,73], it is plausible that the nuclear deformation seen in chronic liver diseases could contribute to HCC development, independent of the underlying etiology.

We have recently reported that nuclear deformation occurs in fatty liver disease, with lipid droplets appearing to cause deformation and compression [75]. Notably, NAFLD patients have an increased risk of HCC development even without cirrhosis [79]. 35–54% of NAFLD-related HCC tumors develop in the absence of cirrhosis, and 18% in the absence of steatohepatitis (NASH) [80,81]. Given the strong association between stiffness and HCC development in cirrhotic livers, we hypothesize that nuclear deformation by lipid droplets could activate cellular mechanosignaling and increase DNA damage, even in a soft liver. We have previously shown that the presence of large lipid droplets in cirrhotic human liver increases the nuclear translocation of YAP, suggesting that nuclear deformation by large droplets may activate mechanosensitive gene expression [75].

## 5. Conclusions

While much cancer mechanobiology research is focused on cancer metastasis or mechanical changes in the tumor microenvironment post oncogenesis, HCC presents a unique case: a primary cancer that occurs 90% of the time in an environment that is already mechanically very stiff. The progression is clear: fibrosis and cirrhosis are associated with large increases in stiffness, further compounded by other associated pressures and mechanical forces, and cancer develops most often in the stiffest livers. Similarly, there is abundant evidence that mechanical changes are associated with malignancy. What has not been shown is that these mechanical changes cause HCC. We hypothesize that liver cells are similar to other cells that undergo malignant transformation in response to mechanical stress: that the pressures and forces experienced by liver cells in the stiff environment of the cirrhotic liver promote genomic instability, nuclear rupture, proliferation, and other oncogenic behaviors. Importantly, recent research suggests that nuclear deformation may serve as a cellular integration point, allowing cells to respond to force regardless of its source. This could further provide an explanation for the rare cases of HCC development in softer fatty liver tissue, whereby intracellular lipid droplets could cause nuclear deformation in a similar manner to increased substrate stiffness.

If mechanical stresses are a source of cancer-causing mutations in hepatocytes, it would offer several new approaches for preventing HCC, including modifying the extracellular environment and altering cell mechanosensitivity. While existing drugs targeting tumor or ECM stiffness have had mixed results in clinical trials, they have been aimed at altering cancer progression rather than development. Treating fibrotic or cirrhotic liver patients before they develop HCC with these drugs as a preventative measure might be more successful.

## Data Availability

Not applicable.
